# The Foreign Journals: Surgery

**Published:** 1841-07

**Authors:** 


*Bulletin de l'Académie Royale de Medécine. Mars* 15, 1841.

### SURGERY.

### *On a new Method of preventing the Entrance of Air into the Chest in the Operation for Empyema.* By M. REYBARD, of Lyons.

IN this proceeding a membranous tube formed of a portion of intestine of a goose is applied by one of its extremities, at the spot selected for puncture, by means of a pellet of diachylon, and communicating by the other with a pig's bladder, the fundus of which gives passage to a trochar, the opening being closed by a ligature. The bladder being folded along the trochar, and the membranous tube firmly supported by the left hand, the trochar is directed along the latter puncture, made in the usual manner, and the trochar withdrawn. Pus runs into the bladder, and gradually distends it. When it ceases to flow, the canula is withdrawn and a ligature applied to the membranous tube, which is cut beyond this point, and the operation is completed by squeezing the diachylon over the wound. The author remarks that this method, with certain modifications, may be used to extract a liquid from any of the large cavities. *Gazette Médicale de Paris*, 3 *Avril*, 1841.

---

### *Section of the Tendons of the Muscles of the Eye in the Horse; Reunion of the Tendons.* By M. LUCIEN BOYER.

AT the sitting of the Royal Academy of Medicine, January 12th, 1841, M. Boyer submitted to the members specimens of pathological anatomy which proved in the most evident manner that when the muscles of the eye have been completely divided, their extremities are not united in any case to each other, but that their reunion to the globe of the eye is effected by an aponeurosis of new formation, which is always inserted behind the primitive insertion on the sclerotic. They also prove that reunion is very quickly effected, and that the muscle, elongated by the interval between its two portions, becomes almost normal in its action. *Journal des Connaisances Médico-Chir. Fevrier*, 1841.

---

### *On Tincture of Iodine as a topical Application to Phagedenic Chancres.* By M. RICORD.

AFTER a trial of all the most common topical applications recommended in the treatment of phagedenic chancre, M. Ricord finds them all frequently inefficacious, and from none has he obtained such prompt and happy results as from the tincture of iodine. He has employed this tincture during the last three months, and almost constantly obtained a prompt modification of the ulcerated surfaces, which soon lose their phagedenic character. This has occurred in many cases. The action of the iodine was particularly evident in a patient who had an open bubo, the ulceration being phagedenic and very extensive. Various means were employed during two months without success, when the tincture of iodine alone so modified the ulcerated surfaces that their extent visibly diminished from day to day. The iodide of potassium had been given in this case without effect, but its use was continued conjointly with the tincture of iodine as a topical application. The cure was perfect in less than a month. *Bulletin Général de Thérapeutique.* 15 *et* 28 *Février*, 1841.

---

*Great Hemorrhage after Division of the Genio-glossi Muscles for Stammering.* By M. Guersent, Jun.

M. Guersent performed the above operation at the Children's Hospital on a child twelve years of age. The hemorrhage at the time of the operation was very slight, but the next day it came on to a large amount, and was checked by charpie dipped in a solution of alum. On the third day the hemorrhage recurred, and was checked by the same means. Fourth day, no hemorrhage, cold lotions, ice in the mouth. Fifth day, hemorrhage came on with the greatest violence, but was stopped by the actual cautery. Sixth day, no hemorrhage. Seventh, it recurred, and the red-hot iron was applied seven times without arresting it; compression, by means of pledgets of charpie steeped in a solution of alum checked it after several hours. The compression was continued on the following day, and permanently suppressed the bleeding. The state of this child excited the greatest solicitude. The weakness and pallor indicated such pressing danger that M. Guersent had determined to tie the lingual arteries if the compression had not effected his object.

*Bulletin Général de Thérapeutique.* *Avril* 15 *et* 30, 1841.

---

*New System of Bandaging.* By M. Rigal, of Gaillac.

The object of this memoir, which was read at the Royal Academy of Medicine in November, 1840, is to point out the advantage obtained by the use of strips of indian rubber in the place of common ligatures and bandages, made elastic by means of the same material, in place of the common roller. The author has used them for the last two years, and speaks very highly of their power of maintaining parts in apposition during the most varied movements of those parts. He particularly instances hare-lip, and the operations for the restitution of lost parts. The elastic bandages are also said to be very useful in maintaining oblique fractures of the lower extremities in perfect apposition, opposing a force in constant operation to the irregular action of the displacing muscles. In this way also they may assist in the replacement of parts after tenotomy.

*Revue Médicale.* *Janvier*, 1841.

---

*Ectropion cured by Autoplasty.* By M. Bérard.

M. Bérard presented to the Academy a young butcher whose inferior eyelid, all except the free border, had been destroyed by a malignant pustule. After having arrested the progress of this terrible disease M. Bérard sought to repair the loss of substance and prevent greater depression of the free border of the eyelid already affected by ectropion. A portion of integument was taken from the temporal region and applied just below the inferior eyelid. The superior border of this slip became united to the inferior border of the eyelid, and its inferior border to the superior edge of the malar integuments. The whole was maintained in apposition by simple agglutinatives. Four months after the operation, the wounds are cicatrized, the new eyelid is perfectly vital, and of a tint precisely similar to that ordinarily seen in the situation which it occupies.

*Bulletin de l'Académie Royale de Médecine.* *Mars* 15, 1841.

### *On Excision of the Elbow-joint.* By M. ROUX.

Since 1812 M. Roux has performed excision of the elbow-joint eleven times, the whole of the articulation having been removed in every case. Of these eleven patients three have died; six reaped every benefit which could arise from the operation. The patient operated on in July, 1839, can flex and extend his arm imperfectly, while a few fistulæ still remain in the patient operated on last November. M. Roux formerly practised the operation of Moreau, making an external and internal vertical incision, and one transverse below the olecranon, but now he only makes an external and a transverse incision, the triangular flaps thus formed being quite sufficient when turned back to expose the articulation for removal, while the subsequent union is much more rapid than when an internal incision is also made.

*Bulletin de l'Académie Royale de Médecine. Mars* 31, 1841.

---

### *On the Impropriety of Dividing the Muscles of the Back in Lateral Curvatures of the Spine.* By M. BOUVIER.

After numerous experiments, M. Bouvier concludes:

1. That the section of the sacro-lumbalis, longissimus dorsi, spino-transverse muscles, &c. is not immediately followed by any diminution of spinal curvature.
2. The changes which the curves undergo during the succeeding mechanical treatment are exactly identical with the changes produced by this treatment alone, when it has not been preceded by the section of the muscles.
3. The space of time necessary to obtain these changes is the same whether we have recourse to orthopedic means alone, or practise also section of the muscles.
4. In a word, dorso-lumbar tenotomy has no kind of influence in remedying lateral deviation of the spine, properly so called.

M. Bouvier further concludes: 1. That the majority of lateral curvatures of the spine are not owing to muscular contraction; and, 2. That etiology, pathological anatomy, and clinical experiments proscribe the section of the muscles of the back in the treatment of these curvature.

*Bulletin de l'Académie Royale de Médecine, Février* 15, 1841.

---

### *Case of Intestinal Invagination in the Cow, cured by Gastrotomy.* By M. BOULEY.

AT the sitting of the Royal Academy of Medicine, March 16, 1841, M. Bouley related a case of intestinal invagination cured by gastrotomy in a heifer. The animal had suffered from colic, and on the third day the symptoms were such as led the veterinary surgeon to suspect internal strangulation. He therefore introduced his arm into the rectum and felt a tumour in the iliac region, which confirmed his diagnosis. The seventh day the symptoms persisting, he performed gastrotomy, guided by the tumour in the iliac fossa, which he then found to consist of five or six pouches of intestine, agglutinated together by effusion from the peritoneum. The operator broke up their adhesions, and withdrew the invaginated portions of intestine by gentle tractor. These portions were perfectly healthy. The opening in the abdominal parietes was united by a suture, and a perfect cure was expected. It may be questioned whether a similar operation would be successful in the horse, as this animal is more liable to peritonitis than the bovine race.

*Gazette Médicale de Paris. Mars* 20, 1841.

---

### *On the Improvement of the Defects in the Refractive Power of the Eye, by Myotomy.* By Dr. KUH, of Breslau.

M. Jules Guerin, Mr. Philipps, and the author have all divided the muscles of the eyeball in the hope of remedying short-sightedness by diminishing the amount of the muscular pressure on the exterior of the eyeball, by which its axis is lengthened. Philipps has divided the inferior oblique, an operation which the author thinks must be perfectly useless. Both he and M. J. Guerin have divided the straight muscles, either the superior and inferior recti, or, in different operations, all the recti of the same eye. The author's first patient, after the division of all the recti muscles, could see to read as well at a distance of nine inches as before the operation he could at a distance of four inches. In the second case, in which the external and internal recti alone were divided the result was null; he could see no better after than before the operation.

*Casper's Wochenschrift. April* 10, 1841.

---

*Report of the Lateral Operations for Stone in Naples, with Statistics of Lithotomy in the last Ten Years.* By M. S. de Renzi.

*First series*—32 men and 1 female operated upon in the year 1839.

Among these 33 patients 10 were under 15 years of age, 22 were adults, 1 was aged. In all there was but a single calculus; 24 were operated on and cured, 9 died. Five were cured in 1 month, 8 in 40 days, 5 in 2 months, 6 in 3 months. Hemorrhage occurred in 5 cases. In 10 the most severe symptoms manifested themselves. In 2 there had been a species of "*pourriture d'hôpital.*" Of the 9 who died, 4 had very large stones, which rendered long and painful manipulations necessary. Of these, 2 died on the second day, 1 on the eighth, 1 on the thirteenth, and all with the symptoms of enteritis or cystitis. Of the other 5, in 1 the dilated bladder filled the pelvis, adhering to the soft parts and the psoas muscle, with an effusion of sanious pus in its cavity. Another, aged 48 years, had purulent collections in the bladder. A child died of vermicular enteritis the third day after operation; 2 others survived 9 and 12 days: and both had purulent collections in the kidneys.

*Second series*—14 men, all operated on with success, 5 of whom were under 15, all the others adults. None of them had dangerous symptoms. In two thirds of these cases the stone was of a moderate size, in the other third it was large.

*Statistics of Lithotomy, from* 1821 *to* 1828, *and during* 1839.

| | Men. | Women. | Cured. | Died. | Children. | Adults. | Old Men. |
|---|---|---|---|---|---|---|---|
| 1821 to 1828 | 579 | 17 | 503 | 91 | 306 | 231 | 59 |
| „ 1839 | 46 | 1 | 38 | 9 | 15 | 31 | 1 |
| | 625 | 18 | | | | | |
| | 643 | | 543 | 100 | 321 | 262 | 60 |

*Il Filiatre Sebezio. Agosto,* 1840.

---

*On the present mode of Treating the Itch at Berlin.* By Dr. Hauck.

The treatment of itch has lately been made the subject of extensive experimental observation in the Berlin hospitals, and it is satisfactory to learn that a slight modification of what is termed the English method, has been found in every respect superior to any other that was adopted, accomplishing as it does all the desirable objects of curing the disease quickly, certainly, and economically.

The remedy employed was the sulphur and soap liniment of the Prussian Military Pharmacopœia, composed of one part of flowers of sulphur, and two parts of soap mixed with sufficient hot water to make them into a soft ointment. The patients after a warm bath of soap and water had been applied, were placed undressed in a chamber, kept constantly at a temperature of 95° Fah., and well rubbed with the ointment over all the parts where the eruption had appeared three times a day, and then made to sweat profusely by putting them into warm beds. This system was continued for three days and nights; on the morning of the fourth each patient had a warm bath, and then if not cured, was provided with clean bed and body-linen, and put in a ward of ordinary temperature, in which the suspicious parts were still rubbed with the ointment, and a warm bath taken every other day. In general, no internal medicines were given; but the diet allowed was reduced to a fourth portion, and water only given to drink.

In this manner, with but one short interval, 1981 were treated and cured between September 1839 and February 1840, making the total number of days of treatment 15,890, which gives on the average 8 days and a small fraction for the cure of each patient, and for the expense of each about two dollars. The exact result was, that

| | | | |
|---|---|---|---|
| In 3 days | there were | cured | 42 |
| — 4 | ,, | ,, | 161 |
| — 5 | ,, | ,, | 333 |
| — 6 | ,, | ,, | 376 |
| — 7 | ,, | ,, | 207 |
| more than 7 | ,, | ,, | 859 |

The treatment of these last was prolonged by many circumstances which can hardly cast discredit on the remedies. In many among them the itch was soon cured, but they remained under treatment for the ulcers which had come on from long neglect of it, or were kept in the hospital till there was no chance of the ulcers communicating the disease. Others among them after being cured of the skin-disease had to be treated for other affections, such as ophthalmia, fever, &c ; and others again had their cure delayed by an obstinate refusal to adopt all accessory treatment. And in addition to these causes giving rise to an apparent increase of the length of time necessary for the cure of the disease, there were some others dependent on the management of the hospitals, and other circumstances quite foreign to the treatment adopted, but which, had they not existed, would have permitted the average number of days of treatment to have been stated much lower.

In the whole 15 months there occurred only 8 cases of relapse, less that is than half per cent. of the cases treated; and among these there was, in many, good reason to suspect a fresh infection. The other cases in which there appeared to be a relapse were in fact only examples of eczema resulting from the stimulus of the skin by the sulphur. In no case did the treatment give rise to any general disorder, or to the inflammations and congestions which some have described as resulting from it.

*Medicinische Zeitung. Februar* 10, 1841.

---

*Cure of an ununited Fracture of the Humerus by a Seton.*
By M. JOBERT, of Lamballe.

A robust man, about forty-five years old, received in a fall a fracture of the humerus, complicated by a small wound at the seat of the fracture. The arm was placed in an immoveable apparatus during a month. At the end of this time the small wound still suppurated, and there was no trace of consolidation. During two months longer the ordinary apparatus was applied, but did not hasten consolidation. M. Jobert then passed a seton between the two fragments, but instead of leaving it five or six weeks, as Physick and others have done, he only let it remain eight days. A month afterwards consolidation was completed. Experience has shown that in some cases, the seton has left after its passage organized fistulæ incapable of cicatrization, which maintain the mobility of the bone Sometimes these passages border on portions of bone denuded by the seton. But when the seton is only left during eight days, it irritates the periosteum, and the inflammation of this latter brings the deposit of a sufficient quantity of osseous matter to effect consolidation.

*Gazette Médicale de Paris.* 5 *Séptembre,* 1840.

*New mode of Treating Orchitis.* By M. Dieulafoy, Surgeon to the Hotel-Dieu at Toulouse.

The following treatment has been employed by this surgeon in more than thirty cases, always with success and without accident: "with the left hand," says he, "I embrace the testicle at its superior part, and thus strongly stretch the scrotum at the inferior part of the organ, and thrust in a very sharp bistoury perpendicularly. Before the instrument is withdrawn it is necessary to enlarge the opening to allow the free exit of fluid. It is necessary to penetrate the tunica vaginalis, or the puncture is without effect. . . . Treated thus the duration of orchitis is from eight to ten days. . . . . I think this is the sole means the surgeon need employ in treating gonorrhœal orchitis. We need not fear to pierce very deeply, and wound the body of the testicle. Galbois has proved that wounds of the body of this organ are not dangerous, and are not followed by any accident."

*Journal de Médecine et de Chirurgie de Toulouse. Juillet,* 1840.

---

*Remarkable Case of Bronchotomy from a Bean in the Air-passages.* By M. Pescheux, of Verneuil.

The principal point of interest in this case is that during this operation a small artery in the neighbourhood of the crico-thyroid membrane was divided, and the child was immediately threatened with suffocation, from the effusion of blood into the trachea. The child voided blood by the mouth and nares, ceased to respire, and became cold, when M. Pescheux instantly applied his mouth to the wound, and sucked out the blood from the trachea for three or four minutes, and being fatigued M. Aury took his place. Some instants afterwards they cauterized the wound and with a female catheter passed into the trachea insufflated the lungs. In a few minutes the child revived, its respiration gradually became regular, and after some cold water had been thrown over it, it looked at the surgeons as though nothing extraordinary had happened. Although the bean could not be found, and was not expelled till five days afterwards, the child perfectly recovered. *Gazette Médicale de Paris.* 29 *Août,* 1840.

---

*Removal of a large part of the Rectum affected with Scirrhus.* By Dr. Francesco Rizzoli, of Bologna.

The patient, Paula Porticelli, was about forty-eight years old when the catamenial discharge permanently disappeared. Numerous hemorrhoidal engorgements supervened, with a sense of pain and weight along the internal part of the sacrum, and constipation, which at length became so obstinate that the patient passed thirteen days without alvine evacuation. There was a discolored fetid exudation from the intestine, and the fæces which came away were in the form of small cylinders. On examining the part scirrhous tubercles were observed, and on passing the finger into the rectum, hard and fungous carcinomata projected, which, about three inches and a half up, were disposed circularly, forming a kind of ring, within which the end of the finger could be introduced with difficulty. An operation being determined on, the patient was placed in bed on the left side, with the thighs bent at right angles with the body, and the nates held apart by an assistant. The operator, with a curved bistoury curved forwards, made a semicircular incision on each side of the anus, which met posteriorly at the coccyx and anteriorly at the perineum; and when the integuments and adipose tissue were divided, he detached the inferior portion of the rectum, taking care to preserve the sphincters. The right index-finger was then introduced above the scirrhous ring, and used to draw it down as far as possible; then the interior portion of the intestine being held to the right and left sides by two assistants with Museaux forceps, and at the same time drawn lower down, Dr. Rizzoli insulated it from the surrounding cellular tissue by the index-finger of the left hand, except where it is connected with the vagina, in which place the union being more intimate and firmer, it was necessary to detach it, partly with the bistoury and partly with a spatula; the finger was then again passed beyond the scirrhous ring, which being used as a guide, by means of a pair of long forceps the intestine was removed by incising it all round, taking care that the edge of the instrument should reach the sound part. After exploring and removing all the diseased parts that remained behind, the bleeding arteries were secured by ligature and torsion, and the venous hemorrhage arrested by means of plugging, and the proper dressings applied. The operation was succeeded in a few hours by fever, which was relieved on the fourth day by five bleedings, and the catheter had to be applied twice. The dressings were changed on the third day, when the patient was again bled, and castor oil given, which procured a copious evacuation followed by great relief. After a few days granulation commenced, and the alvine and vesical evácuations were regular. The patient was attacked with phlebitis on about the thirtieth day, which appeared in both legs, and which gave way to cataplasms and bandaging. Afterwards, the new portion of intestine having a tendency to contract, mechanical dilatation was applied, and the patient continued to live free from any inconvenience and without further medical treatment. *Bulletino delle Scienze Mediche di Bologna. May and June,* 1840.

---

*Notice respecting the Operation for Stammering.*

"To prevent all questions of priority, I hereby inform my colleagues, that I first performed my operation for stammering on the lad Dönau, thirteen years old, on the 7th of January, 1841, and had the honour to present him completely cured at the meeting of the Hufeland Medical Society on the 22d of January, 1841. DIEFFENBACH." *Medicinische Zeitung. Feb.* 3, 1841.

---

*On the subcutaneous Division of the Pronator and other Muscles of the Hands and Fingers.* By Dr. P. DOUBOVITSKI.

THE only point of practical importance in this case, which is related at a most unnecessary length by the patient, who, though a doctor, suffered himself to be grossly maltreated for a fracture through the condyles of the humerus, is that it is not safe to divide tendons that run in synovial sheaths. Two out of four of the tendons of his fingers thus divided failed of reunion, and he lost the power of moving the corresponding phalanges. He knows of other cases in which the same untoward result followed similar operations; and he justly observes that when under such circumstances tendons about the feet have seemed to reunite, it may have been that other muscles have performed the actions that properly belonged to them, and that the success of the operation has consisted, not in the restoration of the action of the contracted muscles, but only in the giving to the distorted foot its proper form.

*Annales de la Chirurgie. Février,* 1841.

---

*On the Treatment of Pseudarthrosis.* By Drs. WOPPISCH and BAUER.

Two cases are here related for the purpose of pointing out the advantages of a long-neglected method of treating false joints, namely, the rubbing together of the ends of the bones. With the exception of the mode of treatment, the cases present nothing of particular interest. In the first, the portions of a fractured thigh which had not united in fifteen weeks were violently rubbed together for a quarter of an hour; the operation gave great pain, but it was followed by such inflammation that the bones united accurately and firmly after a few weeks more. In the second case the fracture (a compound one of the leg) had not united at the end of the eighth week; the same treatment was adopted with an equally favorable result; (but it may be a question whether patience alone might not have obtained the same end.)

*Medicinische Zeitung. December* 30, 1840.

*Iodine in Opacity of the Cornea.* By Dr. Lohsse.

The case in which this remedy was successfully employed was one of opacity of the cornea consequent on syphilitic ophthalmia, and so considerable as almost completely to destroy vision. The iodine was given internally, and from four to six drops of the following collyrium were let fall into each eye three times a day.

℞. Iodini, gr. j.
Potassii iodidi, gr. ij.
Aq. dest. ℥vj. M.

Afterwards this was exchanged for an ointment consisting of iodine, gr. jss, Iodide of potassium, ℈j, and lard, ℥ss, of which a small portion was once or twice a day put between the eyelids. The cure was perfected in three months.

*Medicinische Zeitung. Marz* 3, 1841.

---

*Researches on the Anatomy of the Aponeuroses and Muscles of the Eye, in relation with the cure of Strabismus.* By M. Bonnet, Surgeon to the Hôtel-Dieu at Lyons.

These researches conduce to the scientific explanation of the persistence of the action of the orbital muscles, after the section of their anterior part in the operation for the cure of strabismus; they show the method to be followed in this operation, and throw some light on the movements of the eye and eyelids, considered in the normal state.

The eye is not in contact with the fatty matter of the orbit, as anatomists have stated; it is separated from it by a fibrous capsule, in which it moves with facility. This capsule, concave and open within, is inserted on the anterior extremity of the optic nerve, around the two posterior thirds of the eye without being in contact with them, and terminates on the eyelids on which it is prolonged. The straight and oblique muscles traverse it to reach the eye and contract, with it, intimate adhesions. They have thus two insertions, the one into the sclerotic, the other to the fibrous capsule, and they cannot move one without transmitting to the other all the movements they execute. This has not been hitherto noticed by anatomists, and we shall trace their operation on the movements of the eye and eyelids.

We know that when one of the muscles of the eye has been cut in the operation for strabismus, the increased action which caused the disease immediately ceases, and the movements attributed to the divided muscle are executed as in the healthy state. The explanation of these effects, to be satisfactory, should apply to any of the muscles of the eye indifferently, for after division of any of them the persistence of their function is observed. This cannot be attributed to any phenomenon, which requires, like cicatrization, a work of many days, for the motions effected by the divided muscles are manifested immediately after their section has been made. The anatomical explanation is founded on the fact that the muscles of the eye are inserted both into the sclerotic and the fibrous capsule, and the first of these insertions alone is cut in the operation for strabismus. The second remains entire; the muscle continues to act on the fibrous capsule, and by this medium transmits to the eye these contractions simply weakened.

This double insertion of the muscles of the eye, and the adhesions of this organ to the fibrous capsule explain, it is true, the persistence of the action of the muscles after their division, and indicate the conditions of this persistence, but they do not lead to the knowledge of the method to be adopted in operating for strabismus. But this knowledge is gained in part at least from the dispositions of a fibrous membrane immediately applied over the whole external surface of the sclerotic. This membrane, altogether distinct from the capsule before described, is confounded with the fibrous sheaths of the muscles, and serves to unite them to each other, forming an immediate layer between the conjunctiva and sclerotic. This must be traversed in the operation, and when by its section we have reached the lax cellular tissue which unites it to the eye, the stylet glides without obstacle behind the sheath of the muscles, which, with their aponeurosis, can then be divided certainly and entirely. The knowledge of this membrane gives an astonishing facility to the section of the muscles, both in the living and dead subject, and is as important in the operation for strabismus as that of the sheath of the arteries in the ligature of these vessels.

If it is demanded how the beautiful harmony which always exists between the elevation and depression of the eyelids and similar motions of the eye; or what muscle depresses the lower eyelid; questions hitherto unresolved: their phenomena are readily comprehended when we know that the tarsal cartilages are the continuation of the fibrous capsule into which the latter is inserted, and which conveys the motions of the levator and depressor muscles of the eye. These muscles cannot contract without acting at the same time on both eye and eyelids, and the cause of this simultaneous action is simply anatomical, for we cannot move these muscles in the dead body after having denuded their posterior half, but the eyelids move at the same time and in the same relation with the globe of the eye.

*Bulletin Général de Thérapeutique.* 15 et 28 *Février*, 1841.

---

*On Congenital Dislocation of the Femur.* By M. BOUVIER.

AT the sitting of the Royal Academy of Medicine, Jan. 26, 1841, M. Bouvier presented two preparations illustrative of the two principal forms of congenital dislocation of the femur. In one the head of the femur is received in a superficial cavity above and behind the cotyloid cavity, which latter is reduced to very small dimensions. The capsule embraces both the new and old cavities, and is strongly applied around the femur, the movements of which it limits, and does not allow the head to be brought opposite the cotyloid cavity. The subject was a female seventy-six years of age, and the dislocation was said to have occurred when she was four or five months old, but the probability appeared that it was congenital.

The second specimen was taken from a woman fifty-three years of age, and in this no point of contact exists between the head of the femur and the ilium, which were attached to each other by the capsular ligament and muscles alone. This disposition of parts occurred on both sides, and we know that it is recognized in congenital dislocations alone, and especially in those which affect both sides. The head of the femur is lower and more behind than is usual in iliac luxations, and is only a few lines before the ischiatic notch, and very near the great sciatic nerve. Entirely covered by capsular ligament, the head of the femur rests on a cellulo-fibrous cushion, which here covers the surface of the ilium, and which is united to the capsular ligament by a sort of lax cellular membrane. The round ligament remains, much elongated, flattened, and partly confounded with the capsule. The head of the femur is almost of its normal size, while scarcely a vestige of the cotyloid cavity remains, and this of a triangular form, as is usual in most similar cases. Several of the muscles have undergone important changes, but none in such a manner as to limit the motions of the limb, all of which, with the exception of extension, were almost unaltered.

*Bulletin de l'Académie Royale de Médecine.* 15 *Février*, 1841.

---

*On Compression in the Treatment of Mammary Abscess.*
By MM. TROUSSEAU and CONTOUR.

THIS is a very long paper, illustrated by eight cases, to show the utility of compression of the breast by means of strips of diachylon plaster, an inch broad and several feet in length, carried completely round the body so as to produce regular and methodical compression of the whole breast. The conclusions of the authors are, 1, that this compression may be employed in all forms of inflammation of the breast in nurses; 2, it will sometimes cure when used at the commencement of the inflammation; 3, during suppuration it will not arrest the progress of the formation of pus, but it immediately relieves the pain; 4, compression should be made twenty-four or forty-eight hours after the abscess has been emptied; 5, under its influence the pain ceases, the walls of the abscess unite, fistulæ are cured, and a few days in general complete recovery. Continued for a time it prevents relapse.

*Journal des Connaissances Médico-Chirurgicales. Février*, 1841.

---

*Statistics of Amputation performed in the African Army, in Hospitals and the Field, during the years* 1837-8-9. By Dr. Guyon

The number of amputations performed in the above years (the campaign of Constantine in 1837 excepted,) was 63, namely:

| | |
|---|---|
| Disarticulation of the shoulder-joint | 6 |
| „ „ elbow | 2 |
| „ „ wrist | 6 |
| „ „ knee | 1 |
| „ partial, of foot | 1 |
| „ tarso-metatarsal | 1 |
| Amputation of the thigh | 16 |
| „ „ leg | 7 |
| „ „ arm | 15 |
| „ „ fore-arm | 8 |

Of these 63 patients, 46 were cured, 17 died. As, however, four died from circumstances scarcely connected with the amputation, the proportion of deaths may be stated as 1 to 11. This result is much more favorable than that during the siege of Constantine in 1837, for of 10 amputations performed at Médéah, only 1 survived, and of 62 at Blidah, 39 died.

Of the 63 operations referred to above, 44 were performed immediately, 19 secondarily. The former gave 32 cures, 12 deaths; the latter 14 cures, 5 deaths. Thus the proportion of cures after secondary amputation was not less satisfactory than that after immediate.

*Gazette Médicale de Paris. Février* 13, 1841.

---

*Operations of Laryngotomy*. By M. Guyon.

In a paper on the operations performed with the French army in Africa, M. Guyon states that laryngotomy was twice performed at Bone by M. Génin in croup, "*angines croupales*," but it did not retard death. M. Vital performed the same operation at Constantine for laryngitis, the symptoms of which ceased after its performance. The wound cicatrized, but the same symptoms returned as before, and the patient died six weeks after he had undergone the operation. On post-mortem examination, the mucous membrane was found tumefied so as to obstruct the opening of the larynx. The same operator, at Algiers, performed the same operation to remove a leech from the larynx, and it was crowned with success. M. Guyon states that he and M. Méardi have seen three cases in which a leech had got into the larynx, but they were removed without a necessity for laryngotomy.

*Gazette Médicale de Paris. Avril* 3, 1841.

